# Implementation of a Piezo-diagnostics Approach for Damage Detection Based on PCA in a Linux-Based Embedded Platform

**DOI:** 10.3390/s18113730

**Published:** 2018-11-01

**Authors:** Jhonatan Camacho, Andrés Quintero, Magda Ruiz, Rodolfo Villamizar, Luis Mujica

**Affiliations:** 1 Departament de Matemàtiques, CoDAlab, Escola d’Enginyeria de Barcelona Est (EEBE), Universitat Politècnica de Catalunya, Campus Diagonal-Besòs. C, Eduard Maristany, 6-12, St. Adrià de Besòs, 08930 Barcelona, Spain; jhonatan.camacho@upc.edu (J.C.); magda.ruiz@upc.edu (M.R.); 2 Escuela de Ingenierías Eléctrica, Electrónica y de Telecomunicaciones (E3T), Universidad Industrial de Santander (UIS), Grupo de Control Electrónica Modelado y Simulación (CEMOS), Santander 680002, Colombia; rovillam@uis.edu.co; 3 Centro de Crecimiento Empresarial MacondoLab, Universidad Simón Bolívar (UIS), Barranquilla 080001, Colombia; luis.eduardo.mujica@upc.edu

**Keywords:** principal component analysis, embedded system, online monitoring, structural health monitoring, guided waves, pipeline damage detection

## Abstract

The implementation of damage-detection methods for continuously assessing structural integrity entails systems with attractive features such as storage capabilities, memory capacity, computational complexity and time-consuming processing. In this sense, embedded hardware platforms are a promising technology for developing integrated solutions in Structural Health Monitoring. In this paper, design, test, and specifications for a standalone inspection prototype are presented, which take advantage of piezo-diagnostics principle, statistical processing via Principal Component Analysis (PCA) and embedded systems. The equipment corresponds to a piezoelectric active system with the capability to detect defects in structures, by using a PCA-based algorithm embedded in the Odroid-U3 ARM Linux platform. The operation of the equipment consists of applying, at one side of the structure, wide guided waves by means of piezoelectric devices operated in actuation mode and to record the wave response in another side of the structure by using the same kind of piezoelectric devices operated in sensor mode. Based on the nominal response of the guide wave (no damages), represented by means of a PCA statistical model, the system can detect damages between the actuated/sensed points through squared prediction error (Q-statistical index). The system performance was evaluated in a pipe test bench where two kinds of damages were studied: first, a mass is added to the pipe surface, and then leaks are provoked to the pipe structure by means of a drill tool. The experiments were conducted on two lab structures: (i) a meter carbon-steel pipe section and (ii) a pipe loop structure. The wave response was recorded between the instrumented points for two conditions: (i) The pipe in nominal conditions, where several repetitions will be applied to build the nominal statistical model and (ii) when damage is caused to the pipe (mass adding or leak). Damage conditions were graphically recognized through the Q-statistic chart. Thus, the feasibility to implement an automated real-time diagnostic system is demonstrated with minimum processing resources and hardware flexibility.

## 1. Introduction

Since the construction of buildings, airplanes, and big structures, there has been concern about their condition and how to measure changes over time. To obtain an estimation of these parameters, monitoring systems have been developed on embedded systems. For example, some approaches on Structural Health Monitoring (SHM) have used Wavelet Transform and cross-correlation to detect damage on place using a Digital Signal Processor [1]. PXI platform have been used as well to evaluate structural integrity of high-speed trains and it provided big amounts of data from brake, engine start and operation states [2]. Some platforms such as the one proposed in Ref. [3] have budget constraints for providing vibration information using a DAQ system. Yan et al. [4] proposed a technique to detect cracks on concrete structures using NI CompactRIO platform and a FPGA to validate damage localization algorithms was used by Liu and Yuan [5]. Some commercial solutions for SHM have been developed by companies such as Accelent with their Portable ScanGenie that integrated with Layer Sensors and Smartpatch (their operative software) and can be used to monitor engine disks, joints, beams among other objects [6]. Digitexx Data System [7] also provides semi-permanent solutions for SHMstructural health monitoring, providing data from many different sensors such as accelerometers and tiny manometers.

However, these systems have several implementation problems since they have not demonstrated high reliability and feasibility [8]. Regarding this concern, the piezo-diagnostics principle has proved to be useful to detect the elastic wave’s reflections caused by discontinuities like boundaries or other type of damages [9,10,11,12]. Kobaldi et al. [13] use a method to simulate a cracked pipeline and, with some piezoelectric sensors and actuators, obtains a bond graph. On the other hand, PCAprincipal component analysis has been reported to be effective for addressing feature extraction and pattern recognition tasks in methodologies implementing monitoring systems for SHM [14,15,16]. Also, the effectiveness of using PCA algorithm in combination with piezo-diagnostics principle to detect structural damages has been demonstrated in the author’s previous works to be promising approach in order to detect and locate damages on different type of structures as pipes, wind turbines, and aircraft sections, among others [17,18,19,20].

Thus, by combining Principal Component Analysis (PCA) with the piezo-diagnostics principle coded in an embedded system, advantages such as risk reduction in the loss of information are added. In addition, greater flexibility regarding information accessibility and storing is provided, facilitating making decisions to ensure structural health, as some experts in remote monitoring can assure [21]. In this work, a preliminary embedded system is built, where most problems relate to computational resources, such as memory and processor performance, are no longer a major problem for structural damage assessment. In addition, several desirable features for condition monitoring systems such as user-friendly results interpretation, low power requirements, easy setup, low cost, small size, expandability and hardware accessibility can be obtained. Thus, the successful application of PCA-based approaches have motivated the integration of this technology with the help of embedded hardware.

This paper presents an implementation of a piezo-diagnostics approach for damage detection, using PCA in a Linux-based embedded platform Odroid-U3. To detect structural damages, the methodology consists firstly of obtaining a structural baseline model by applying PCA on a set of experiments from pristine condition of the structure. Then, current condition (Damaged or Undamaged) of the structure is evaluated by comparing new measurements with respect to the baseline model [22]. The following elements are shown: instrumentation equipment, computational core capability, programming procedure, and practical considerations by considering statistical indexes provided by [23].

The feasibility of the system was verified by conducting lab experiments in a pipe section in order to detect reversible damage provoked by adding a mass. Also, leak conditions were studied in a pipe loop to evaluate the monitoring capacity of the system. Thus, the capability of the system for online and automated structural health monitoring through the implementation of embedded approach of PCA-SHM algorithms is demonstrated.

## 2. Architecture of the Proposed Piezo-Diagnostics System

**Figure 1:**
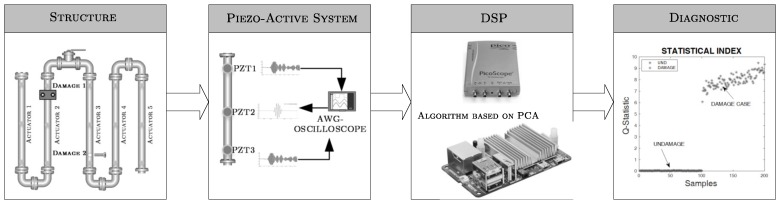
Damage diagnosis system schematic.

By considering the elastic wave propagation principle, an equipment was developed for condition monitoring, whose architecture is schematized in Figure 1. Its main components are: (i) the structure to be monitored, (ii) piezoelectric devices attached to the structure surface, (iii) excitation elements, (iv) power supply, (v) data acquisition components, (vi) digital processing unit (Odroid-U3) and (vii) means to show results.

**Figure 2:**
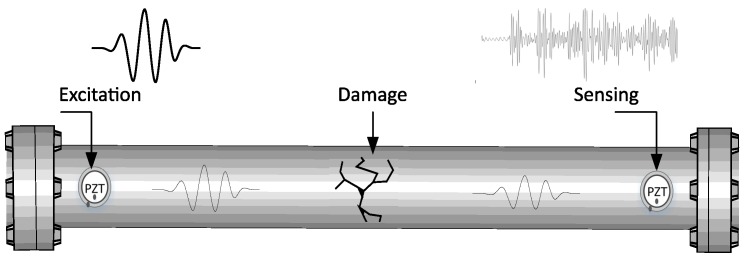
Piezo-diagnostics principle [26].

The equipment uses an active piezo-diagnosis scheme that consists of using piezoelectric devices to produce elastic waves and evaluate its propagation along the examined structure. Figure 2 presents the specific configuration used here used, where a PZT mounted on the structure surface operates as an actuator (electrical energy is converted to mechanical energy is converted to produce guided waves along the surface) by taking advantage of piezoelectric effect. The other PZT works as a sensors in a pitch-catch mode to measure elastic wave propagation, that contains information about scattering, reflection, and mode conversion. The applicability of PCA statistical modeling on guided waves response is demonstrated in author’s previous works [24,25], where it is evaluated the feasibility of a monitoring scheme based on torsional guided waves for detecting damages in pipes is evaluated. Thus, the acoustoelasticity effect is implemented taking advantage of a data-driven approach based on statistical processing for structural monitoring purposes.

The most important parameters to be considered for generating guided waves are related to frequency and type of electric field excitation, coupling material for the bonding layer, and recommendations for electrical connection of piezoelectric elements. Thus, the following experimentation conditions should be considered:

- A burst-type signal is applied to obtain a PZT’s actuator response near its resonance frequency (around 100 Khz). Since, this type of signal has finite duration and contains frequency components around a central band, a maximum amplitude can be guaranteed during the process.
- Adhesive Cyanoacrylate is used as coupling layer, which has a better performance than other materials due to repeatability of its waveform pattern and transmitted energy [27]. Also, the adhesive property makes it suitable for continuous monitoring tasks, unlike other materials used for ultrasonic tests.
- General soldering procedure was done using APC instructions [28]. Due to some tests executed, it is recommended to use shielded and twisted pair wires to cancel external noise and it is highly recommended to build a circuit that works as interface between piezo-devices and electronic components.

## 3. Implemented Piezo-Diagnosis Methodology

This chapter describes the Piezo-diagnostics methodology implemented in the Odroid-U3 Linux-based embedded platform and presents PCA foundations used in it. The methodology for embedding the damage-detection algorithm based on PCA is detailed, with special focus on programming procedures to manage computational requirements according to ARM systems capacity. Also, instrumentation and hardware facilities are detailed, which allows the overall integration of the PCA-based piezo-diagnostic technology.

### 3.1. PCA Foundations

PCArincipal Component Analysis (PCA) mathematical background has been covered by many authors and books, thus only a brief description is shown to provide insight of the overall technique used by the equipment. Let us assume that it is necessary to describe a 3d object using its projections only, so the best course of action is to find the better angle of the object that provides more information without the need for more projections. Mathematically speaking, first it is necessary to build a matrix X with n rows and m columns containing information from n trials and m sensors. Then, the data matrix X should be standardized before applying PCA, using mean and standard deviation of all measurements from sensors to eliminate differences between variable ranges. Afterwards, the covariance matrix must be calculated by the following formula previously to obtain the projections:

(1) $$C_{\tilde{x}}=\frac{1}{n-1}{\tilde{X}}^{t}\tilde{X}$$

$C_{\tilde{x}}$ is a square symmetric matrix of dimension *m* that measures the extent of the linear relationship between every pair of variables on the data set and *t* denotes the transpose operation. Principal Components of data matrix *X* are obtained from solving the eigenvalue decomposition problem in terms of $C_{\tilde{x}}$. The eigenvectors with the highest eigenvalues are the principal components of the data set. By ordering the eigenvalues, their order of significance can be obtained through the total variance of data set. By keeping a desired number of eigenvectors, *r*, a feature vector $\varphi_{r}$ (matrix of vectors) can be formed to represent most of the data variance. The new matrix $\varphi_{r}$ together with standardization parameters can be called a PCA model of the original data set. Geometrically speaking, the PCA model allows the projection of the original data over principal components which is a minimized variance representation [29].

### 3.2. PCA-Based Algorithm

Based on the advantages of PCA technique, an algorithm was proposed to create statistical models and to extract features of structural response for being compared with a nominal state. These statistical models are obtained by computing from PCA, transformation and normalization matrices and the principal component’s variances vector for guided-wave responses of no damage cases. After this process, Q-statistic is used as detection index to compare respect to baseline-line performance (PCA model of no damage state). This process can be done for different damage conditions, providing different insights on structural response. Two stages are necessary to obtain a performable version of the algorithm: PCA model building (Training Stage) and PCA features extraction for Structural Condition Monitoring (Monitoring Stage).

#### 3.2.1. Training Stage: Model Building

**Figure 3:**
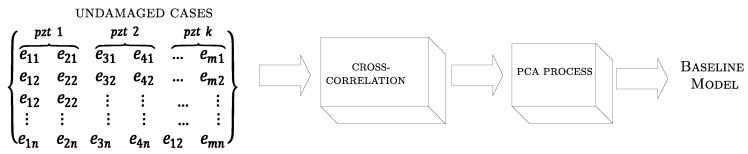
PCA training diagram.

Since the implemented algorithm needs an undamaged structural responses case matrix, it is necessary to obtain a PCA model of it, which is the baseline performance. The matrix is arranged such that each column contains piezoelectric time samples due to guided-wave response (collected over a specific time period from different sensors installed at crucial points of structure), while each row represents a new independent piezoelectric response for the same undamaged structure. The undamaged case matrix is represented by first block of Figure 3.

Once an undamaged case matrix is built, a PCA model is obtained by following the next steps:

- Normalize the undamaged cases matrix by using the mean values ${\hat{\mu }}_{i}$ and standard deviations ${\hat{\sigma }}_{i}$ of undamaged case matrix according to group-scaling method [22]. Therefore, each data-point is scaled by considering changes between sensors. As a result, from the standardization, k− standard deviations and *m*-mean values are obtained, where *k* is the number of PZT sensors and *m* corresponds to the total variables analyzed (m=N∗k, N are the number of recorded samples per sensor which depends of the sample time).
- Estimate the reduced principal component matrix by obtaining the eigenvectors $\varphi_{r}\rfloor^{Und}$ and eigenvalues $\lambda_{i}$ of the covariance matrix using an iterative or low-cost computational method [22,30,31].
- Keeppt only the most *r* representative principal components in terms of the data variance and reconstruction error [29]. We recommend using the same number of principal components than the number of experiments.

The final PCA model coded in the embedded system to compute online features of each piezoelectric response, contains the next three elements:

- The normalization matrix represented by mean and deviation values of undamaged cases matrix.
- The transformation matrix represented by the eigenvectors
- The principal components variances represented by the eigenvalues

To eliminate noisy data trends and exclude common external signal, a preprocessing stage based on cross-correlation analysis is implemented. Thus, cross-correlation between actuation and sensing piezo-signals is computed before applying PCA.

#### 3.2.2. Monitoring Stage

To validate the presence or absence of damage in the structure, new PZT measurements are processed. Thus, a row vector is organized with current PZT measurements and a Q-statistic is implemented to identify deviations from the undamaged PCA statistical model. The Q-values chart is obtained by computing the squared prediction error resulting from the reconstruction with the PCA model. Q-index has shown to be successful in fault diagnosis systems, it allows monitoring differences between baseline and current state, which are attributed to damage. Also, it is suitable for visualization purposes on 2D plots of different structural damage conditions and it is easier adapted as inputs for supervised and unsupervised algorithms to obtain complementary results regarding to damage classification and quantification tasks [32,33]. To compute Q, a projection (scores) of the current normalized data onto the PCA space is obtained, by using Equation (2).

(2) $$T=\varphi_{r}\rfloor^{Und}*\tilde{x};\;\tilde{x}=\left(\frac{x-{\hat{\mu }}_{i}}{{\hat{\sigma }}_{i}}\right)$$

where *x* is a vector with PZT measurements from current condition of the structure, $\tilde{x}$ is the normalized PZT measurements vector, is the projection of the normalized measurements onto the PCA model, and $\varphi_{r}\rfloor^{Und}$ are the *r* principal components obtained from normalized undamaged matrix. Finally, the Q-statistics index is obtained by using Equation (3).

(3) $$Q=e^{t}e;\;e=\tilde{x}-{\left[\varphi_{r}\rfloor^{Und}\right]}^{t}*T$$

where *t* denotes the transpose operation, and *e* is the projection onto the residual left components and it represents the reconstruction error.

## 4. Hardware Design

**Figure 4:**
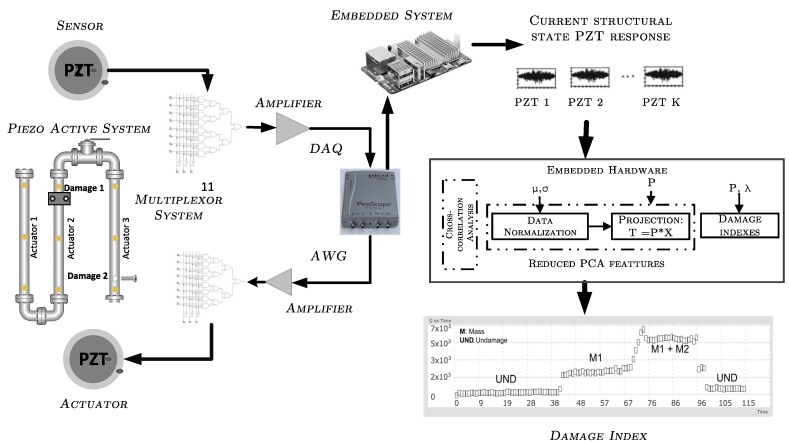
Logical relationship configuration diagram.

The system configuration and its electronic components is depicted in Figure 4, where its main component is the embedded platform (system’s core) capable of streaming, processing (mathematical computations) and storing data, as well as handling a user interface. Other system components are the data acquisition system, amplifiers, and signals conditioners.

### 4.1. Signal Conditioning and Acquisition System

**Figure 5:**
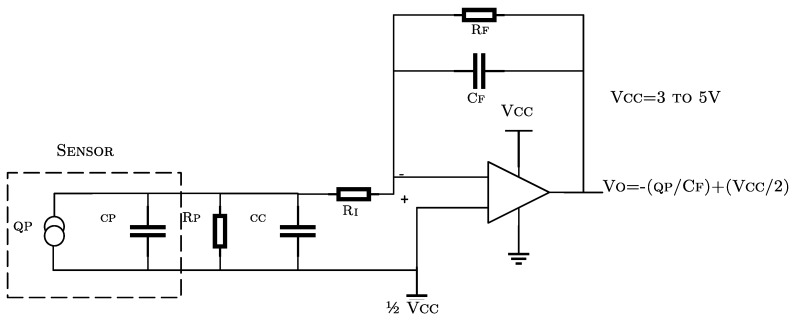
Charge amplifier [34].

It was necessary to build a signal conditioning circuit for electrical coupling between PZT devices and electronic components, where a charge mode amplifier was used to ensure the operating frequency and to minimize signal loss due to loading effect [34] (see Figure 5). Piezo electric response is amplified to ±10 V and acquired by means of a Picoscope 2000 and a 16-channel multiplexor board, such that each PZT response can be acquired in each channel with low delay, depending on the total amount of sensors connected. The PicoScope™ includes Arbitrary Wave Generation (AWG) function, which allows generate burst excitation. It is highlighted that PicoScope™ is used as DAQ/Generation system since it has desirable features for standalone and portable systems: Good bandwidth, faster waveform update rates, low price (From $129), and ultra-compact size compared to other commercial devices [35]. The sample rate of system is conditioned by the acquisition system bandwidth (100 MHZ) and the number of PZT-sensor channels (16). Thus, the maximum achievable sampling frequency in the system is 6.25 MS/s.

### 4.2. Embedded Platform

An embedded system is a small device designed to execute specific tasks and contains elements that vary according to its goal, but it always maintains a group of basic elements for operation: RAM memory, input-output peripherals and a microprocessor as CPU. These platforms are generally based on ARM architectures, with smaller size than regular computers making them ideal for low power applications. One of its main features is its capability of processing information in real-time. A good platform for SHM is Odroid-U3 and its characteristics are summarized in Table 1.

According to Table 1, the Odroid-U3 has a peripherals package that allows making several improvements using Ethernet communication, USB, SD, HDMI ports, video out and on-board memory. It also allows execution in real time of operating system such as Ubuntu Distributions, which supports the architecture of the system described in this paper. The final component included in the hardware system design corresponds to the USB-to-IO expansion board that provides GPIO interface. It is used to implement the logical programming of the multiplexor board through the PIC18F45K50 microcontroller.

## 5. Algorithm Programming

**Figure 6:**
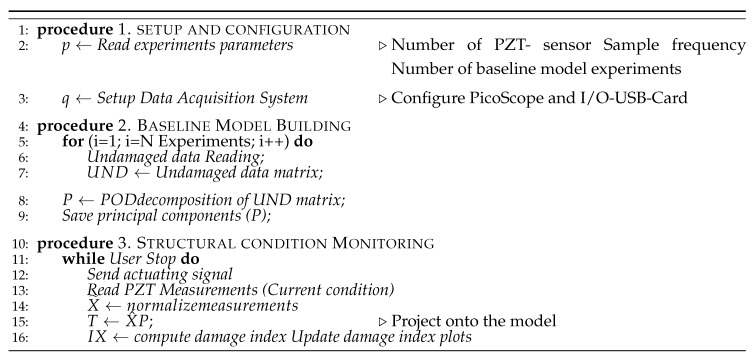
PCA-Based Piezodiagnostic Damage-Detection Algorithm.

The overall methodology described in Section 3.2 is implemented in the embedded hardware by using the flowchart detailed in Figure 6, which is designed to evaluate the current time structural state through index damage charts.

According to Figure 6, the embedded algorithm consists of three procedures: set up and configuration, baseline model building (training stage) and structural condition monitoring (monitoring stage), which are sequentially executed. Once the configuration parameters and drivers to manage data acquisition are established, the transformation matrix P of the statistical model is obtained via Proper Orthogonal Decomposition method (POD) [30]. POD require fewer computational resources over alternative methods such as SVD and NIPALS, maintaining a compromise among resources memory and time consumptioning [20]. Thus, POD method is implemented as processing tool for baseline model building since low memory and execution time are required.

## 6. Results and Validation

**Figure 7:**
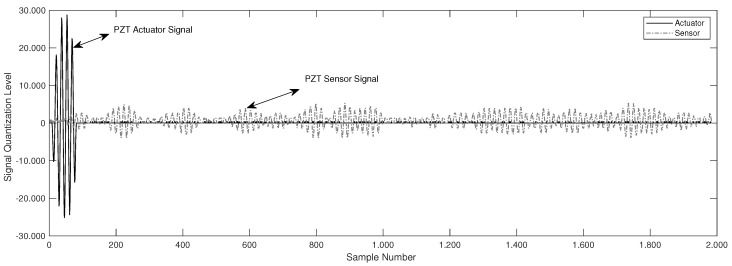
Actuation and sensing signals.

This section details the main features and performance of the integrated piezoelectric damage diagnosis system. Validation tests were conducted on two structural lab models to evaluate the system performance: (i) a carbon-steel pipe section, and (ii) a pipe loop bench. These two experiments were configured to evaluate the performance of the system and the overall methodology. The first experiment was validated in a pipe section with reversible damages by adding masses to the surface structure and the second one corresponds to a carbon-steel pipe loop configured to study leaks type damage. For both experiments guided waves was induced with 5 cycles of burst-type pulse. One example of the scattered waves recovered is illustrated in Figure 7.

For all experiments, baseline model was obtained by applying PCA to 100-experiment repetitions during 1 s of periodic excitation signal (Ts=40 ns).

### 6.1. Piezo-Diagnoster Hardware Performance

**Figure 8:**
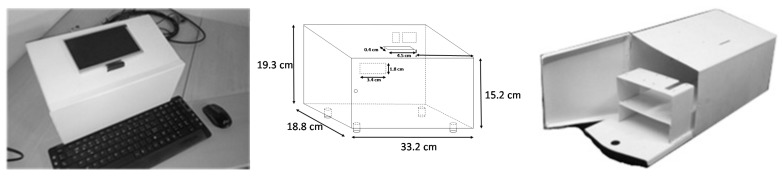
Piezo-diagnoster hardware platform.

The piezo-diagnoster equipment developed for standalone inspection tasks is detailed in Figure 8. Its dimensions are 19.3 cm height × 18.8 cm large × 33.2 cm width and it is provided with a 7” HDMI display of 800 × 480 resolution. Configuration and operation are achieved by using standard input/output peripherals (keyboard and mouse).

**Figure 9:**
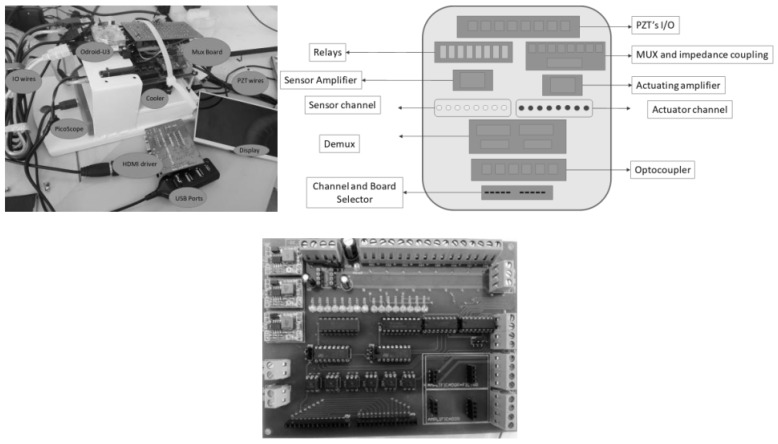
Components of Piezo-diagnoster hardware platform.

The instrumentation components including the data acquisition system, amplifiers and signal conditioners are detailed in Figure 9. They comprise peripheral packages for Ethernet communication, as well as USB, SD, HDMI ports, video out and on-board memory.

The system is able to acquire signals up to a maximum of 24 PZT sensors, whose hardware was designed with J-fet technology amplifiers (THS4031) that allows a proper bandwidth response (100 MHZ), high switching speed and good signal/noise ratio. Voltage gain is adjustable in order to manage drifts and offset of different structures to be monitored, which is produced by electrical and mechanical coupling effects.

The typical performance of the proposed piezo-diagnoster system for a set of 8 PZT sensors is summarized in Table 2.

Results in Table 2 regarding to time corresponds to mean, while memory resources to maximum peak from all processed data in real time. A significant delay is observed in the performance of the system due mainly to graphical tasks, thus a dedicated graphical unit is required to manage visualization process. Likewise, results storage is managed through unformatted text-file writing and should be considered as resource demanding task. According to Table 2, the embedded hardware platform accomplishes the computing tasks with acceptable resource consumption for monitoring purposes. A comparative test was conducted in a general-purpose computer (Intel core I5 @2.67 GHz and 3.8 GB RAM) where results was obtained in 0.4219993 s; demonstrating the consistency of the embedded algorithm with minimal hardware resources.

### 6.2. Reversible Damage Assessment in a Pipe Section

**Figure 10:**
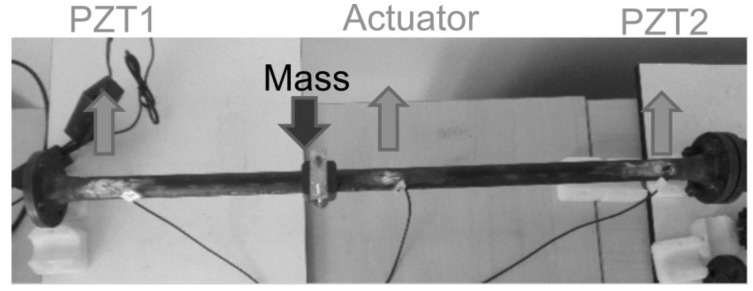
Pipe Section experiment mockup.

A carbon-steel pipe section test bench was used to validate the system performance. The pipe section (Figure 10) is 100 cm length × 2.54 cm diameter × 0.3 cm thickness and contains bridles at its ends. Three piezoelectric devices were attached to the surface of the structure, where the PZT located at the middle was used as actuator element and those near to the bridles as sensors. Acquisition hardware was configured in single mode setup; thus, a pitch-catch record is obtained from the respective PZT- sensor each time that an actuation signal is sent to PZT actuator. As it is illustrated in Figure 10, a special shaped accessory was added to the surface pipe section in order to recreate damages of type mass adding at different locations of the surface.

**Figure 11:**
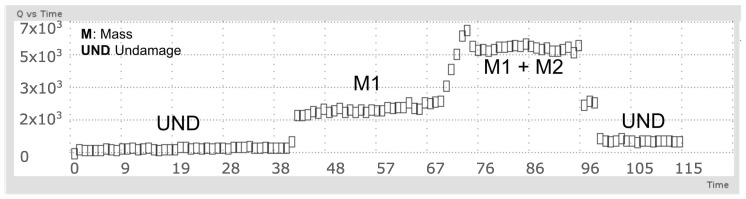
Mass adding damage detection for a pipe section.

The behavior of Q-index when the mass element is added to the surface structure is presented in Figure 11. First, the structure is monitored without damage, then the special mass accessory was added between PZT1 and PZT actuator (M1), next and additional mass was added between PZT2 and PZT actuator (M1 + M2) and finally both masses was removed to return to the initial undamaged condition.

**Figure 12:**
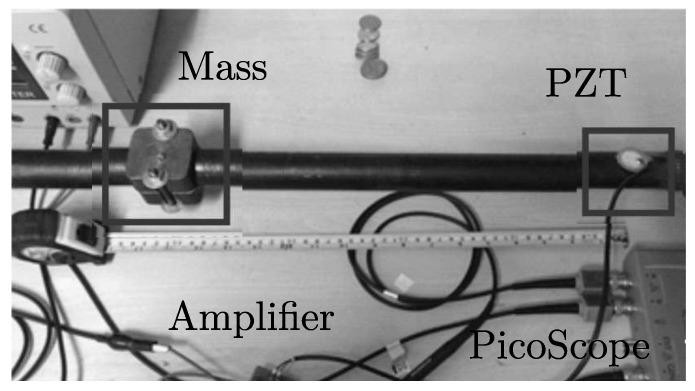
Mass displacement experiment mockup.

According to Figure 11, a clear difference between undamaged and damaged conditions is observed for abrupt changes in Q-values, and minimal variations are obtained for healthy state of the structure. Also, transient response is captured while the masses are being installed. A second experiment was conducted to evaluate the system response sensitivity by locating the mass (damage) at different points of the test specimen. In this case, two piezoelectric devices (sensor-actuator) were attached near to the structure bridles and 100 experiments per condition (Damaged/Undamaged) were registered. Fifteen damage scenarios, (denominated D1,D2,⋯,D15), were recreated locating the mass at 5 cm, 10 cm, and so on, respect to the PZT actuator (see Figure 12).

**Figure 13:**
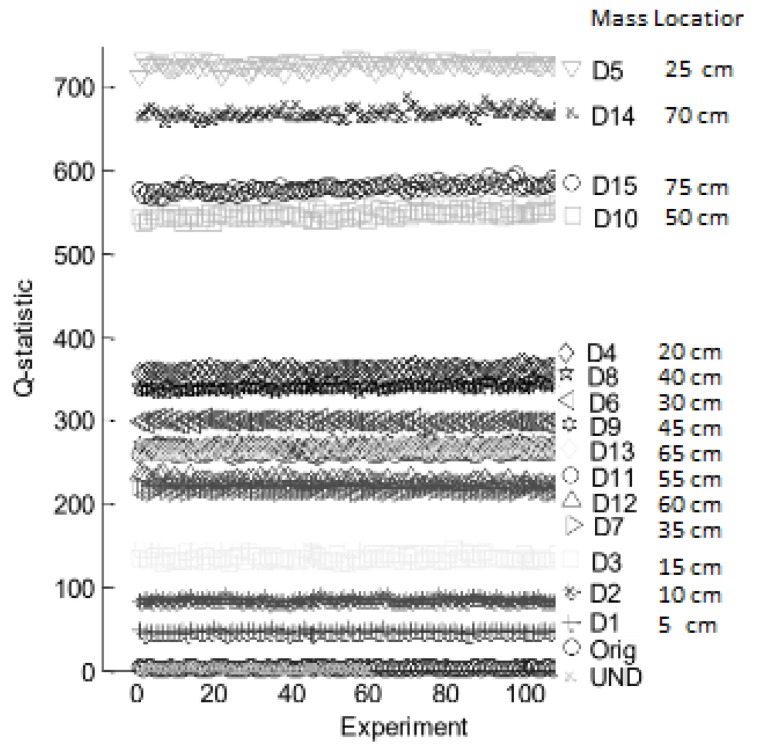
Sensitivity of Q-statistical index for mass location experiment.

Performance of Q-statistic damage index is shown in Figure 13, where all damage conditions are summarized in a scatter plot with ordered labels according to ascending Q-values for a better visual interpretation. Experiments related to pristine structure cases, used for validation purposes, are labeled as ‘UND’ and those used to build the PCA model as ‘Orig’.

According to results in Figure 13, the system differentiates between damaged condition and healthy state, maintaining repeatability in Q-values for pristine condition (Orig and UND). However, some scenarios are confused despite boundaries between several damage experiments. This overlapping is detailed in Table 3, where it is observed that this occurs for experiments recorded when the mass is located around the middle of the pipe section, with major influence just in the center of the structure. For scenarios away from the center of the pipe better decision margins was obtained.

Also, the Q-values are not ordered in relation to mass position, meaning that is not possible use Q-statistic as measurement of damage location.

### 6.3. Leak Detection in a Pipe Loop

**Figure 14:**
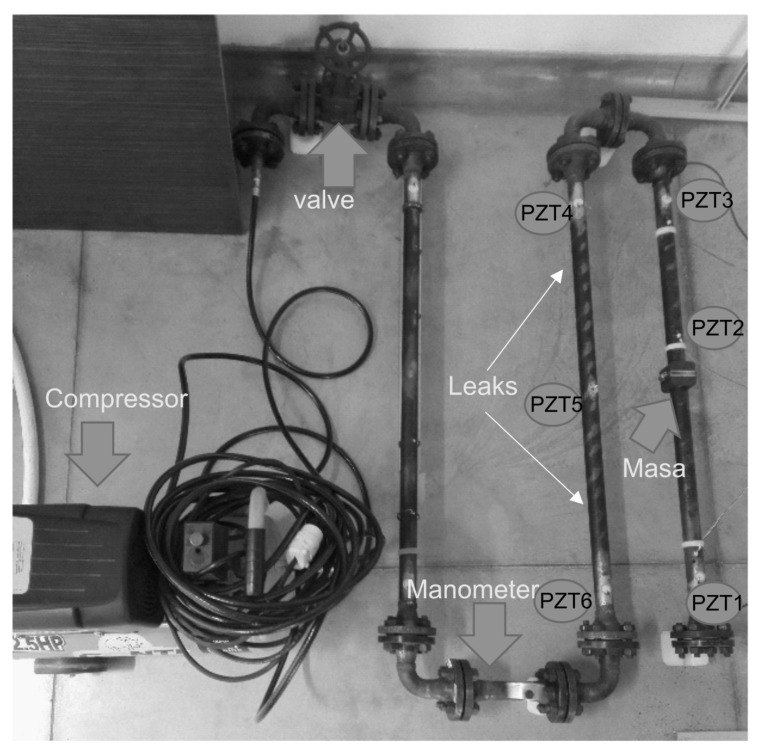
Pipe loop experiment.

Figure 14 shows the experiment configuration for leak detection, which consists of three, 100 cm length × 2.54 cm diameter × 0.3 cm thickness, carbon-steel pipe sections. Each pipe section contains bridles at its ends and three piezoelectric devices (PZT) bonded along the surface structure. The PZT devices located at the middle of each section operate as actuators and the remaining ones as sensors. A valve controls airflow at 80 psi from a compressor installed in the pipe loop, while a manometer indicates the operation pressure. Acquisition hardware was configured in pairwise mode setup, such that sequential pitch-catch records are obtained from each pipe section in single mode set up.

**Figure 15:**
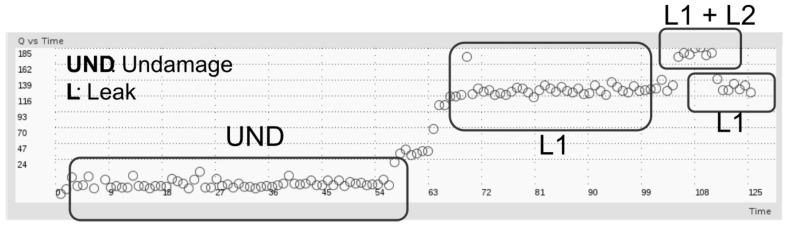
Leak detection in the loop experiment.

As it is illustrated in Figure 14, leaks were produced by a full opening of a hole between the PZT devices (Actuator-Sensor) and located at different points along the structure. These kinds of leaks are recreated by means of 1/4−inch holes drilled along the pipe section wall, maintaining the pipe loop pressure at 80 psi. In this case, the influence of damages at curve portion of the pipe were not studied and no PZT devices were located there. However, every pipe section is considered to be part of the undamaged state for baseline model building, by concatenating the respective PZT response of each section in the undamaged case matrix. Figure 15 presents the corresponding Q-values for leak damage cases. First, the pipe loop is monitored in healthy state (no damage), then a leak is caused in one section (L1), next an additional leak is recreated in other section (L1 + L2) and finally the last leak is plugged with a Teflon screw returning to condition where only one leak is present (L1).

The results presented in Figure 15 confirm the suitability of Q-index for differentiating between damaged and undamaged conditions. In addition, the performance of Q-index shows that the system has capability for detecting different damage types (mass and leaks), high sensitivity to transient response and capacity to recover a previous state or condition. A final remark is highlighted about differentiation between leak and mass-adding damage types, which requires additional modeling stages such as neural networks and complementary features as it is demonstrated in previous works [36]. Thus, by using only Q-index it is not possible to distinguish between mass damage and leak.

## 7. Conclusions

The feasibility for real-time monitoring of structural condition was demonstrated by embedding a PCA-based piezo-diagnostics approach in the Odroid-U3 ARM platform. The robustness of the system was experimentally validated demonstrating its capacity to detect different damage conditions such as leaks and mass adding in pipe structures. Continuous monitoring is achieved by implementing squared prediction error (Q-statistic) as index to identify deviations from undamaged structural state. Thus, a qualitative damage measurement through Q-index was obtained, where the presence or absence of damage can be related to changes in Q-values. However, future works to determine the non-linear relationship between weight difference and damage extent should be conducted. Thereby, it was demonstrated the applicability of embedded systems to develop technology for its application in the practical engineering field and monitoring equipment for research use in SHM applications was demonstrated. Future works are recommended regarding the implementation of methods for damage location and quantification in order toto improve the capability of the system including the detection of damages in areas of difficult access such as the curved portion of a pipe. Also, it is suggested to conduct experiments by using other structures and to evaluate evaluation of the robustness for variable operational and environmental conditions is suggested. Finally, some additional tests should be conducted considering aging properties of the whole elements included in the system.

## Figures and Tables

**Table 1:** Odroid-U3 characteristics.

Feature	Description
CPU	1.7 GHz Exynos4412 Prime Cortex-A9 Quad-core processor
	2Gbyte LPDDR2 880Mega Data Rate
SO	ubuntu-14.04.2lts-lubuntu-odroid-u-20150224
RAM	2072 [MB]
On-boardboard Flash	8 Gb, eMMC
Power Source	5VDC/2A
USB 2.0 Host	3 × USB 2.0, 1 × Micro USB
Serial Port	UART 1.8 V
Ethernet	10/100, RJ45
Video Out	HDMI (480p/720p/1080p)
GPIO	5

**Table 2:** Average performance of Piezo-diagnoster system.

	CPU Usage %	Memory Usage %	Time Response	Visualization Delay
Graphical interface	10.2	2.1	∼1 s	4 s
PCA processing	50.3	0.9	0.879 s	NA
TOTAL	60.5	3.0	1.876 s	

**Table 3:** Overlap degree of mass location experiment.

Group	Damage Labels	Mass Location [cm]	Overlapping
0	{Orig, UND}	No damage	Expected
1	{D7, D12}	[35, 60]	Minimal
2	{D11, D13, D9}	[55, 65, 45]	Full
3	{D8, D4}	[40, 20]	Minimal
